# Infection with *Babesia canis* in dogs in the Algiers region: Parasitological and serological study

**DOI:** 10.14202/vetworld.2020.1351-1357

**Published:** 2020-07-15

**Authors:** Amel Kiouani, N. Azzag, S. Tennah, F. Ghalmi

**Affiliations:** Laboratory of Research Management of Local Animal Resources (GRAL), National Veterinary College of Algiers, Road Issad Abbes, El Alia, Algiers, Algeria

**Keywords:** Algiers, *Babesia canis*, *Babesia* spp, blood smears, dogs, prevalence, risk factors, serology, ticks

## Abstract

**Background and Aim:**

Canine babesiosis is a vector-borne disease transmitted by ticks of the Ixodidae family. The effects of infection in dogs can range from the subclinical to the severe lethal form. This study aimed to make an original contribution to the knowledge of circulating species of *Babesia* spp. in dogs in the region of Algiers as well as mechanisms and risk factors for their transmission.

**Materials and Methods:**

An epidemiological study was carried out on 189 blood samples taken from dogs from April 2015 to January 2016. The samples taken underwent parasitological by Giemsa-stained blood smear and serological analyzes by indirect fluorescent antibody test (IFAT). The ticks were looked on all the dogs taken.

**Results:**

Giemsa-stained blood smears revealed the presence of two groups of parasites of the genus *Babesia*: Large *Babesia* (3/25, 12%) and small *Babesia* (22/25, 88%). The IFAT at a dilution of 1/32 showed an overall seroprevalence with *Babesia canis* of 17.98% (95% confidence interval 11.53-22.46). The distribution of the antibody titers for the positive samples showed that of the 34 positive sera with a titer ≥1/32, 28 sera remained positive at a dilution of 1/64 (14.81%), 22 at a dilution of 1/128 (11.64%) and 15 sera remained positive at a dilution of 1/256 (7.93%). Although seroprevalence varied according to canine population (20% and 19.49% in pet dogs and canine pound dogs, respectively, and 6.66-0% in farm dogs and hunting dogs, respectively), statistical analysis showed no significant differences between populations. The antibody titers obtained after several dilutions showed that 22 canine pound dog sera remained positive at a dilution of 1/128 compared to pet dogs and farm dogs which ceased to be positive at the dilution of 1/64. The comparison between the two diagnostic methods showed a strong agreement between the parasitological examination by blood smear and the serological method by IFAT. However, IFAT was much more sensitive. The analysis of risk factors, which may influence *B. canis* seroprevalence, has shown the influence of age, tick presence, and season. Finally, of the 242 ticks collected from a total of 59 dogs, only one tick species was identified *, Rhipicephalus sanguineus*.

**Conclusion:**

This study indicates a frequent circulation of species of *Babesia* in the dog in the Algiers region and *R. sanguineus* was the only tick identified.

## Introduction

Canine babesiosis is a vector-borne disease transmitted by ticks of the family Ixodidae, representing a major problem of veterinary interest, and it is caused by an intraerythrocytic protozoan of the genus *Babesia* and *Theileria* affecting the dog worldwide [1], the infection in dogs can vary from a simple subclinical form to a severe and sometimes deadly form [2]. The classic form is characterized by a combination of febrile syndrome with a hemolytic state, sometimes evolving toward severe renal insufficiency or even a fatal shock [3]. This diversity is mainly related to the species of *Babesia* involved, the age of the animal, its immune and physiological status, and the abundance of infected ticks [4]. Usually, the diagnosis of *Babesia* infection is based on the morphological characters of the intraerythrocytic forms observed on a peripheral blood smear [5]. In the dog, *Babesia canis* (large *Babesia*) and *Babesia* *gibsoni* (small *Babesia*) have long been considered the only species described that cause canine babesiosis anywhere in the world [6]. However, based on differences in antigenic properties as well as in the geographical and specific distribution of the vector, it has been proposed that *B. canis* can be divided into three subspecies: *Babesia canis canis* transmitted by *Dermacentor reticulatus*, *Babesia canis vogeli* transmitted by *Rhipicephalus sanguineus*, and *Babesia canis rossi* transmitted by *Haemaphysalis leachi* [7,8]. More recently, with the advent of molecular phylogenetic analysis, in particular genotyping of the small ribosomal subunit 18S gene, it was concluded that these subspecies are actually distinct species, named *B. canis*, *B. rossi*, and *B. vogeli* [9]. The spectrum of *Babesia* species of small pathogenic forms that infect dogs has increased in recent years and their diversity has been greater than expected. In fact, in addition to *B. gibsoni*, two genetically and clinically different species are currently described that cause diseases in dogs: *Babesia conradae*, identified in dogs in the West US [10] and *Theileria annae*, described as a piroplasm close to the species *Babesia* *microti* [11].

Babesiosis is one of the most important tick-borne infectious diseases of domestic and wild mammals and still poses significant diagnostic and therapeutic challenges for veterinary practitioners around the world, and it is an increasing problem worldwide due to the expansion of tick habitats and the increased mobility of animals, which promote the spread of parasites into new geographical areas [12].

In Algeria, infection with *Babesia* spp. is most commonly diagnosed in dogs by morphological identification of intraerythrocytic piroplasm from peripheral blood smear. To date, very little knowledge is available on its distribution and actual prevalence, as well as on the nature of the risk factors determining its transmission. This study aimed to make an original contribution to the knowledge of circulating species of *Babesia* spp. in dogs in the region of Algiers as well as mechanisms and risk factors for their transmission.

## Materials and Methods

### Ethical approval

All procedures performed in this study, including the collection of blood sample and ticks, were in accordance with the animal use in research of National Veterinary College of Algiers.

### Sampling and study area

Our study was conducted in the region of Algiers, which is located at the edge of the Mediterranean, it occupies a central position in the North of Algeria and it has a Mediterranean climate. Between April 2015 and January 2016, 189 dogs from Algiers region were tested for the presence of *Babesia* parasites. The dogs are divided into four different populations (10 pet dogs 5.4%, 159 canine pound dogs 84%, 15 farm dogs 8%, and 5 hunting dogs 2.6%) of different breeds, age (young <6 months, adult), and both genders.

### Laboratory analyses

For each animal, a thin blood smears were prepared from peripheral blood, fixed with methanol, stained with Giemsa solution, and then examined under light microscopy (×100) for the detection of possible intraerythrocytic piroplasm.

Blood samples were collected in a dry tube for sera collection and analyzed by indirect fluorescent antibody test (IFAT) “kit MegaScreen FLUOBABESIA canis” by serial dilution (1/32, 1/64, 1/128, and 1/256) according to the manufacturer’s instructions; then, the slides were examined under a fluorescence microscope.

Ticks were searched on all sampled dogs. Each tick was identified (genus, species, and gender) by binocular magnification (OPTIKA).

### Statistical analysis

The calculated prevalences were estimated at 95% confidence interval. Statistical differences in proportions were compared using the Chi-square test (Yates corrected). The observed differences were considered significant when p<0.05 was obtained. A comparison between tests was calculated by kappa Cohen methods with the calculation of specificity, sensitivity, accuracy, and Cohen’s kappa.

## Results

### Blood smear

Of 189 dogs, *Babesia* spp. were detected in 25 (13.22%), no significant difference was observed for different dog populations, although the dog pound was more infected than other dog populations, two groups of *Babesia* were identified, (3/25) large *Babesia* (12%) and (22/25) small *Babesia* 88%.

### Serologic test

Of a total of 189 sera tested by IFAT at a dilution of 1/32, 34 were found to be positive for antibodies specific to *B. canis* (17.98%). The observed seroprevalences did not differ significantly between the different populations. Although the lowest seroprevalences were observed in hunting dogs (0%) and farm dogs (6.66%), the highest were observed in stray (19.49%) and companion dogs (20%) (Table-1).

**Table-1:** Prevalence of anti-*Babesia canis* antibodies in different canine populations.

Canine population	Negative	Positive	Total	Prevalence % (IC 95%)
Canine pound	128	31	159	19.49% (12.77-25.22)
Pet	08	2	10	20% (0-45.25)
Farm	14	1	15	6.66% (0-18)
Hunting	5	0	5	0%
Total	155	34	189	17.98% (11.53-22.46)

The prevalence of antibody titers obtained after several dilutions, the highest levels of antibodies were observed in canine pound dog (22 sera remained positive at a dilution of 1/128 compared to pet dogs and farm dogs that have ceased to be positive at 1/64 dilution) (Table-2).

**Table-2:** Distribution of antibodies titer anti-*Babesia canis* in function of dogs’ populations.

Dogs titers	Canine pound	Pet	Hunting	Farm	Total positive
32	31	2	0	1	34
64	25/31	2	0	1	28/34
128	22/31	0	0	0	22/34
256	15/31	0	0	0	15/34

### Comparison of the two methods of diagnosis

The performance of the blood smear method was evaluated by calculating Cohen’s sensitivity, specificity, relative accuracy, and kappa coefficient against the IFAT serological test at a titer (≥1/32) taken as a reference test for the diagnosis of *B. canis* (Table-3). For the results of the blood smear, we only considered the positive smears for large *Babesia* (3/25) corresponding to *B. canis*.

**Table-3:** Comparison of the BS technique with the IFAT (titer ≥1/32) as a reference test for the diagnosis of *Babesia canis.*

Tests for the diagnosis of *B. canis*	IFAT			

		+	−	Total
Blood smears (3/25) large Babesia	+	1	2	3
	−	33	153	186
	Total Intrinsic values	34	155	189
		Se=3%	Sp=99%	RA=81%
	Kappa=0.80			

Se=Sensitivity, Sp=Specificity, RA=Accuracy, IFAT=Indirect fluorescent antibody test

The blood smear method showed good specificity 99%, very low sensitivity of 3%, and an accuracy of 81% compared to the IFAT.

The calculation of a coefficient k (0, 80) indicates a strong agreement between the two methods.

### Risk factors

This study, based on the results of the seroprevalence study, allowed us to identify the risk factors that seem to increase the risk of being seropositive to *B. canis*. Table-4 summarizes all the results obtained from the analysis of the risk factors presumed related to infection with *B. canis*.

**Table-4:** Analysis of some potential risk factors that may influence the seroprevalence of *Babesia canis*.

Variables	n	Number of positives	Seroprevalence (%) (95% CI)	p-value
Gender				
Female	77	10	12.98 (4.59-19.40)	
Male	112	24	21.42 (13.30-28.69)	>0.1
Age (months)				
<6	67	7	10.44 (2.66-17.33)	<0.05
>6	122	27	22.13 (14.49-29.50)	
Ticks				
Presence	59	19	32.20 (19.85-44.14)	
Absence	130	15	11.53 (5.5-16.48)	<0.0001
Season				
Autumn	60	3	5 (0-10.62)	
Summer	47	9	19.14 (7.55-30.44)	
Winter	28	0	0	<0.0001
Spring	54	22	40.74 (26.66-53.33)	

No significant difference was observed in prevalence between genders of dogs for *B. canis* (p>0.05). On the other hand, a significant variation was observed according to the age category of the animals (p<0.05). Youngsters <6 months old seem less infected (10.44%) than adult dogs more than 6 months old (22.13%). Thus, it can be seen that the proportion of infected dogs increases with age.

Seroprevalence also varied depending on the presence or absence of ticks on the animal; dogs with ticks are more infected than dogs without ticks (p<0.001).

The season has a very significant influence on the seroprevalence of *B. canis* (p<0.001). The infection rate is significantly higher in the spring 40.74 (26.66-53.33) followed by the summer 19.14 (7.55-30.44).

### Epidemiological study of tick populations encountered in dogs in the study area

Of the 189 dogs examined, 59 were infested with the tick *R. sanguineus*, an overall prevalence of 32.20%. The total number of ticks collected was 242 ticks, divided into 56 males and 146 females, 36 nymphs, and 4 larvae. We have studied some risk factors that may positively or negatively influence the infestation of dog by tick, the results reveal that only factor of the season which have been shown to be significantly associated with the presence of ticks (p<0.05) (Table-5).

**Table-5:** Analysis of risk factors that may influence infestation of dog by ticks.

Variables	n	Number of positives	Infestation rate by ticks (%) (95% CI)	p-value
Gender				
Female	77	23	29.87 (18.65-39.34)	
Male	112	36	32.14 (23.18-40.81)	0.9
Age (months)				
<6	67	20	29.85 (17.91-40.08)	0.9
>6	122	39	31.96 (22.62-39.37)	
Season				
Autumn	60	1	1.66 (0.00-3)	
Summer	47	19	40.4 (25.70-54.29)	<0.0001
Winter	28	0	0	
Spring	54	39	72.22 (59.77-84.22)	
Canine population				
Canine Pound	159	48	30.18 (22.73-37.26)	0.2
Pet	10	5	50 (18.37-81.62)	
Hunting	5	0	0	
Farm	15	6	40 (14.70-65.29)	

## Discussion

### Prevalence of infections with Babesia spp. using the blood smear method

Of 189 dogs, *Babesia* spp. were detected in 25 (13.22%), which is slightly higher than that reported in India (7.47%) [13] and in Albania (9%) [14]. No significant difference was observed for different dog populations, although the dog pound was more infected than other dog populations, two groups of the genus *Babesia* were identified, large *Babesia* (12%) which is higher than those reported in India (0.93%) [15] and in Pakistan (5%) [16], on the other hand, was fairly close to that reported in South Africa (11.69%) [17].

Another study in Algeria at El Taref region has obtained a higher prevalence for large *Babesia* (30%) than ours [18], 22 dogs were found positive to small *Babesia* among dogs positive to *Babesia* spp. with a prevalence of 88%, which is higher than that obtained in Serbia (1.7%) [19] and in India (7.9%) [20], (35.13%) [15]. On the other hand, our prevalence was close enough of that reported in South India (84.9%) [21].

### Seroprevalence to infections with *B. canis* using the IFAT

Of a total of 189 sera tested by IFAT at a dilution of 1/32, 34 were found to be positive for antibodies specific to *B. canis*, which corresponded to a seroprevalence of 17.98%. This result was close to that reported in Romania (19.8%) [22] by the same technique and same dilution. The seroprevalence obtained in our study is much lower than that obtained in Italy in 2012 (70%) [23] but clearly higher than those reported in Italy in 2016 (8.8%) [24], in Hungary (5.7%) [25], and in Slovakia (8.4%) [26], this diversity of results between different regions of the world can be attributed to the climatic conditions that influence the geographical distribution of vector ticks in the transmission of *B. canis*.

In this study, several dog populations with varying degrees of exposure were analyzed. The observed seroprevalences did not differ significantly between the different populations. However, work reported in Romania showed significant differences in the seroprevalence between rural dogs and urban dogs. In fact, rural dogs had the highest rates [27]. The absence of a significant difference in the seroprevalence between the different canine populations can be related to the sampling carried out. Indeed, our sampling is characterized by heterogeneity and disparity in numbers, with a significantly higher sampling rate (84%) in canine pound dogs compared to other populations (hunting dogs, farm dogs, and pet dogs) with 8%, 5.3%, and 2.6%, respectively.

Another point worthy of mention is that of the frequency distribution of antibody titers. In fact, the antibody titers obtained after several dilutions, showed that the serological test detected high levels of antibodies for dogs in the canine pound (22 sera remained positive at a dilution of 1/128 compared to pet dogs and farm dogs that have ceased to be positive at 1/64 dilution). This result suggests that the canine pound dogs are in constant contact with the source of infection. As a result, they can spread the infection to ticks, increasing the risk of infection.

### Comparison of the two methods of diagnosis

The blood smear method showed good specificity 99%, very low sensitivity of 3%, and an accuracy of 81% compared to the IFAT at a threshold titer (≥1/32). The calculation of the agreement between the two methods by the use of Cohen’s kappa test showed a coefficient k of 0.80, which corresponds to a strong agreement between the two methods. This lack of sensitivity observed for the blood smear would, therefore, explain the huge difference obtained between the parasitological and serological results. In Italy, a similar study was carried out by evaluating the blood smear parasitological method using the IFAT as a reference in the diagnosis of *B. canis*. They also showed that IFAT generated more positives (34%) compared to blood smear (2.4%). However, the Cohen kappa coefficient gave a value of 0.019, indicating a very low agreement between the two tests [28]. The low susceptibility of a blood smear to serologic tests can be explained by the low parasitemia that characterizes the chronic carriage of these diseases [29], and on the other hand, the serological method can detect animals that have been in contact with the parasite whose IgG was detectable up to 420 days’ post-infection [30]. However, the cross-reactions are commonly reported in IFAT [31]. As a result, the detection of chronic and subclinical babesiosis in carrier dogs requires molecular tools [9].

### Associated risk factors

Several authors around the world have studied the risk factors associated with canine babesiosis. No significant difference could be demonstrated between the seropositivity of males and females for *B. canis*, and this seems compatible with the results obtained in India [32] and Romania [22]. However, other studies show that the physiological state of females can influence the degree of sensitivity of the animals. In fact, pregnant and lactating females have a higher sensitivity since maternity gives rise to a state of immunodepression, favorable to the development of the disease [33]. A significant variation was observed according to the age category of the animals. Young dog under 6 months of age seems less infected than adult dogs over 6 months old. Thus, we found that the proportion of infected dogs increases with age, this is consistent with the results obtained in South Italy [34], contrary to the results of another study that shows that young dogs are more sensitive than adults [24]. Some studies have shown no significant age-related differences [24,34]. The low rate of infestation in young dogs can be explained by their low chance of becoming infected or developing a detectable immune response [21].

Seroprevalence also varied depending on the presence or absence of ticks on the animal, dogs with ticks are more infected than dogs without ticks; this seems compatible with the results obtained in Nigeria [35].

The Mediterranean climate is favorable for some tick species, such as *R. sanguineus*. Data from the literature have shown the role of this species in the transmission of *B. canis* [28,35], it is more abundant during the dry season, during which the prevalence of canine babesiosis is very high [36]. The region of Algiers benefits from a Mediterranean climate, and it has significantly influenced the seroprevalence of *B. canis*. Indeed, the infection rate is significantly higher in spring, followed by summer. Our results support those obtained in Zambia, which show a high prevalence of *B. canis* during the months of May, June, and July [37]. According to the literature, babesiosis can occur during autumn and spring, thanks to favorable conditions for the multiplication of ticks responsible for the transmission of *B. canis* [33].

### Epidemiological study of tick populations encountered in dogs in the study area

Of the 189 dogs examined, 59 were infested with the tick *R. sanguineus*, an overall prevalence of 32.20%. Our results are in agreement with those obtained in Palestine [38], Central America [39], and Australia [40]. On the other hand, *D. reticulatus* is a known vector of *B. canis* in the UK [41] and Croatia [42] and *Ixodes ricinus* is the vector of *Babesia* spp. in Romania [43].

The total number of ticks collected was 242 ticks, divided into 56 males and 146 females *R. sanguineus*, 36 nymphs, and 4 larvae of the genus *Rhipicephalus*. We have studied some risk factors that may positively or negatively influence infestation of dog by tick. The results showed that only season factor was significantly associated with the presence of ticks. Our result about season is in agreement with those obtained in Central Europe [44].

Our results show that pet dogs are the most infested by ticks; contrary to data of literature who indicate that infestation with ticks is related to the lifestyle of dogs, it is usually stray dogs that are most at risk of contact with ticks [33], this can be explained by sampling. Indeed, our sampling is characterized by heterogeneity and a disparity in the workforce; the collection rate in the group of companion and farm dogs is very low compared to dogs in the canine pound. The prevalence of tick-infested dogs is very high during the spring. This is in line with the results of the seroprevalence study, which indicated that the season factor is strongly correlated with seropositivity rates to *B. canis*.

## Conclusion

This work allowed us to highlight by parasitological examination *Babesia* spp. and to estimate by serological analysis the seroprevalence of the anti-*B. canis* antibodies in different canine populations of the Algiers region, which indicates a frequent circulation of this species of *Babesia* in the dog in this region. On the other hand, we have identified the ticks collected from some dogs, the species *R. sanguineus* (adults, nymph, and larva) was identified.

## Authors’ Contributions

AK, NA, and FG conceptualized and designed this research. AK collected blood samples, ticks and drafted the first version of manuscript. AK, NA, ST and FG analyzed data and results. NA, ST, and FG revised and finalized the manuscript. All authors have read and approved the final manuscript.
